# A retrospective study of antipsychotic drug switching in a pediatric population

**DOI:** 10.1186/1471-244X-13-248

**Published:** 2013-10-08

**Authors:** David Linton, Ric M Procyshyn, Dean Elbe, Lik Hang N Lee, Alasdair M Barr

**Affiliations:** 1 British Columbia Mental Health and Addictions Services Research Institute, 938 W 28th Avenue, Vancouver, BC V5Z 4H4, Canada; 2 Department of Anesthesiology, Pharmacology & Therapeutics, University of British Columbia, Vancouver V6T 1Z3, Canada; 3 Department of Psychiatry, University of British Columbia, Vancouver V6T 1Z3, Canada

**Keywords:** Adolescent, Antipsychotic, Children, Drug, Polypharmacy, Switching

## Abstract

**Background:**

Antipsychotic drugs can be used to help treat a wide variety of psychiatric disorders. However, specific antipsychotic drugs for any particular patient may need to be changed for a number of different reasons, including a lack of therapeutic efficacy and / or intolerance to medication side-effects. Drug switching may occur through a limited number of established patterns. The nature of these changes is not well characterized in youth, despite their frequent occurrence.

**Methods:**

A retrospective analysis of antipsychotic drug switches was conducted on patients who had been admitted as inpatients to a tertiary care child and adolescent psychiatric institute. PharmaNet (a large, central administrative database) records of all medications prescribed in the 52 weeks prior to admission, and then between admission and discharge, were analyzed for switching patterns. Additional data regarding diagnoses were obtained from medical chart review.

**Results:**

Patients represented a diagnostically heterogeneous population, and almost all antipsychotic drugs were administered off-label. In the one year prior to and during admission to the hospital, a total of 31 out of 139 patients switched antipsychotic drugs. The frequency of switching increased closer to the time of admission, and the proportional rate of switching was even higher during hospital stay. The most common switch was from risperidone to quetiapine. Our analysis identified three main patterns of drug switching, all occurring with similar frequency: titrated drug switches, abrupt drug switches and concurrent drug administration.

**Conclusions:**

The present study indicates that antipsychotic drug switching in youth may be relatively common, particularly in the year prior to hospitalization. No specific manner of drug switching predominates. This study also demonstrates the feasibility of using large administrative databases to characterise switching patterns in youth.

## Background

Due in large part to the advent of the second-generation antipsychotic drugs that are associated with fewer neurological side-effects than their predecessors, there has been a widespread recent increase in the utilization of antipsychotic drugs. This has included the expansion of their use to include disorders other than psychosis, for both on- and off-label indications [1]. One patient population than has experienced a particularly rapid increase in the rate of treatment with second-generation antipsychotic drugs is youth. Rates of antipsychotic drug use among children and adolescents are at historic highs [2,3], and this includes a large proportion of patients treated for off-label indications [4-6]. Studies of this population have extensively described the patterns and treatment strategies for antipsychotic drug use in youth: topics range from clinical efficacy [7-9] through to drug associated side-effects [10-12]. However, almost no research has focused on the nature of switching from one antipsychotic drug to another, which is a common and important consideration in the treatment of children and adolescents with second-generation antipsychotic drugs.

Switching between antipsychotic drugs may occur for a number of different reasons, in both the youth and adult populations. One of the most frequently cited causes, regardless of indication, relates to a lack of clinical efficacy [13]. A failure of the patient to respond adequately, and for the drug to show minimal impact on psychiatric symptoms, represents a major rationale for antipsychotic drug switching. Similarly, a lack of tolerance to second-generation antipsychotic drug-associated side-effects, which typically include cardio-metabolic sequelae [14-18], may result in a change of antipsychotic medication [19]. Interestingly, a retrospective study by Nyhius and colleagues [20] of antipsychotic drug switching in the adult population found that the two strongest predictors of switching early during antipsychotic drug treatment related to a worsening of akathisia and an increase in depression/anxiety symptoms.

In adults, not only the drugs which are used during the switch itself, but also the manner in which a switch is managed, can strongly increase the risk of developing additional adverse effects, that may include symptom relapse [21,22]. Despite these risks, there are often benefits conferred by selecting an alternative antipsychotic medication [23]. Switching antipsychotic medications happens frequently in adults, with two U.S. studies providing estimates of between 29.5% [20] and 42% [24] of patients treated with antipsychotics switching drugs per year. Although the risk of rebound or relapse effects has been noted in poorly managed switches [25], we were unable, in a thorough search of the literature, to identify previous studies that had systematically described the prevalence of different techniques of switching antipsychotic drugs (although see [26]). In addition, there is also an absence of data describing the frequency and the nature of antipsychotic drug switches in juveniles.

In the present study, we therefore evaluated the frequency and patterns of antipsychotic drug switching in youth in the year preceding their admission to a tertiary care psychiatric institute. Our data consist of prescriptions made to inpatients on the psychiatric wards of a pediatric tertiary care facility in Vancouver, British Columbia, as well as all prescriptions made to these patients during the 52-week period prior to their admission. At this tertiary care facility, patients treated with antipsychotic drugs represented a wide range of different psychiatric disorders, and as such are a heterogeneous population. Off-label use of antipsychotics in youth is common in this population, which includes disorders other than psychosis. In large part , this is because antipsychotic drugs are used to treat symptoms that are common across multiple diagnostic boundaries, such as behavioral dysregulation [27]. We used a simple and novel analysis to identify and characterize switches between antipsychotic medications. Due to the nature of our data, it was also possible to examine when, relative to the date of admission, switches were performed, and the prevalence of different switching methods. Additionally, we describe the choices of drugs that were used in this cohort, both in the community and following admission to the hospital.

## Methods

The present study represents a retrospective analysis of data from all patients, aged 18 years and under, who were admitted to the psychiatric inpatient program at the tertiary care BC Children’s Hospital in Vancouver, British Columbia, Canada. Patients were identified based on admission to one of the psychiatric wards at the B.C. Children’s Hospital between May 1, 2008 and December 31, 2009. The Child and Youth Mental Health Program at B.C. Children’s Hospital is a Provincial resource providing mental health assessment and specialized treatment for the entire Province. It is also the Province’s main treatment, research and teaching facility for youth mental health. The study protocol was approved by the University of British Columbia Research Ethics Board. The study was conducted in accordance with the principles of Good Clinical Practices and the Declaration of Helsinki.

To investigate psychotropic medication use in the 52 weeks prior to hospital admission, data were obtained for prescriptions during this period through B.C. PharmaNet, which is a Province-wide network that links all pharmacies in B.C. to a central set of data systems [28] and can provide medication records as far back as 14 months. PharmaNet records contain information including the prescribing physician, name and manufacturer of the drug, quantity dispensed, dose and directions for the patient. To investigate medications prescribed during hospitalization, which includes the entire period between admission and discharge, data were abstracted from the Department of Pharmacy’s computer database (GE Centricity Pharmacy, v8.2). Data collected from B.C. PharmaNet and the Department of Pharmacy included the following list of medications: antipsychotics, antidepressants, mood stabilizers, benzodiazepines, anticholinergics, stimulants, and sleep medications. This method did not include “as needed” *hora somni* medications. A total of 335 patients were identified based on their admission date to the B.C. Children’s Hospital; 302 of these patients had complete information. These records were used to characterize modifications made to antipsychotic therapy (including starting antipsychotic therapy, discontinuation of antipsychotic therapy and switching antipsychotic medications), and the drugs, cross-tapering strategies, and doses involved in antipsychotic switches.

“Starts” were defined as a new prescription recorded after at least 3 weeks with no previous antipsychotic drug prescription. Similarly, “stops” were defined as a discontinuation of an antipsychotic drug for at least 3 weeks, without a new prescription of another antipsychotic medication. “Switches” were defined as when one antipsychotic was terminated and a different antipsychotic was started within at least 3 weeks, or if the two antipsychotic prescriptions overlapped with a titration down (eventually ending in termination) within 3 weeks. Switches were classified into one of four categories, based on previously defined approaches: [26,29,30] (i) titrated switches where at least one drug was tapered, (ii) immediate switches where the first drug was terminated and the second drug started abruptly with no overlap or tapering, (iii)“gap” switches, with a gap of less than 3 weeks between antipsychotic prescriptions, and (iv) “overlapped” switches where both the initial and the new antipsychotic were given concurrently for some period of less than three weeks ending in a termination of the first antipsychotic with no titration down. Psychiatric diagnoses represent diagnoses made by physicians at the time of discharge from the hospital. All psychiatric diagnoses were obtained from electronic mental health records or physician’s notes within the patient’s medical chart.

## Results

In the year prior to hospital admission, there were 4818 weeks of antipsychotic prescription which were given to a total of 139 patients. Following hospital admission there were 315.4 weeks of prescriptions recorded. The mean age of patients was 12.9 ± 3.0 years, and the population was 63% male. Primary psychiatric diagnosis at discharge was attention deficit hyperactivity disorder in 35 patients, anxiety in 35 patients, depressive disorder in 19 patients, autism in 18 patients, psychosis in 14 patients, bipolar disorder in 12 patients and tic/neurological disorder in 2 patients (Table 1). Antipsychotic drug dosing, based on chlorpromazine equivalents, differed significantly between diagnoses [F(_7_,_137_) = 5.86, p < 0.0001)], with dosing notably lower in the two largest treatment group of ADHD and anxiety patients than for patients with depression, bipolar disorder or psychosis. Antipsychotic polypharmacy (i.e., concurrent administration of two or more antipsychotic drugs) was noted in 35 patients during the study period. Psychotherapeutic drug combination therapy was relatively common. Of the 139 patients receiving antipsychotic treatment, 56 patients were concurrently treated at some point with a psychostimulant/ADHD medication, 70 patients with an antidepressant, 33 patients with a mood stabilizer, 11 with a benzodiazepine, and 39 with any other class of psychotropic drug (such as a sleep medication). By comparison, only two patients were treated with a diabetic medication, and 12 were prescribed an antihypertensive agent.

**Table 1:** Characterization of study population receiving treatment with an antipsychotic medication

	**Depressive disorder**	**Anxiety**	**Attention-deficit hyperactivity disorder**	**Psychosis**	**Bipolar disorder**	**Substance abuse**	**Autism**	**Tics/Neurological disorder**
Subjects (M/F)	19 (6/13)	35 (27/8)	35 (26/9)	14 (8/6)	12 (6/6)	3 (2/1)	18 (12/6)	2 (2/0)
Mean Age (SD)	15.05 (1.92)	11.10 (2.81)	10.45 (2.60)	15.65 (1.25)	13.07 (3.22)	15.76 (0.54)	12.42 (3.17)	10.41 (2.21)
Risperidone (% of Dx group)	3 (15.8)	14 (40.0)	17 (48.6)	5 (35.7)	1 (8.3)	0 (0.0)	7 (38.9)	0 (0.0)
Mean Dose mg (SD)	0.92 (0.63)	0.89 (0.50)	1.01 (0.50)	2.20 (1.10)	1.00	0	1.54 (1.10)	0
Olanzapine (% of Dx group)	5 (26.3)	0 (0.0)	0 (0.0)	5 (35.7)	0 (0.0)	0 (0.0)	3 (16.7)	0 (0.0)
Mean Dose mg (SD)	6.00 (1.37)	0	0	15.00 (3.54)	0	0	11.67 (2.89)	0
Quetiapine (% of Dx group)	7 (36.8)	11 (31.4)	7 (20.0)	5 (35.7)	8 (66.7)	0 (0.0)	5 (27.8)	1 (50.0)
Mean Dose mg (SD)	251.79 (195.31)	138.64 (128.51)	89.29 (34.93)	325.00 (269.26)	276.56 (208.14)	0	160.00 (96.18)	75
Ziprasidone (% of Dx group)	0 (0.0)	0 (0.0)	1 (2.9)	1 (7.1)	0 (0.0)	0 (0.0)	0 (0.0)	0 (0.0)
Mean Dose mg (SD)	0	0	40	20	0	0	0	0
Aripiprazole (% of Dx group)	0 (0.0)	0 (0.0)	0 (0.0)	1 (7.1)	0 (0.0)	0 (0.0)	0 (0.0)	0 (0.0)
Mean Dose mg (SD)	0	0	0	7.5	0	0	0	0
FGAs (% of Dx group)	2 (10.5)	0 (0.0)	0 (0.0)	2 (14.3)	0 (0.0)	0 (0.0)	1 (5.6)	0 (0.0)
Mean Dose mg (SD)	-	-	-	-	-	-	-	-
CPZ Equiv mg (SD)	220.60 (217.14)	110.76 (134.21)	73.44 (54.30)	316.07 (253.32)	333.33 (280.50)	0	186.01 (185.73)	100.00
# Emergency Visits (SD)	0.58 (0.77)	0.66 (1.37)	0.74 (1.77)	0.79 (1.19)	0.33 (0.78)	1.67 (1.15)	0.78 (2.07)	1.50 (2.12)
Switched Pre Hosp (% of Dx group)	7 (36.8)	5 (14.3)	6 (17.1)	1 (7.1)	3 (25.0)	1 (33.3)	2 (11.1)	1 (50.0)
Switched Post Hosp (% of Dx group)	0 (0.0)	0 (0.0)	1 (2.9)	4 (28.6)	1 (8.3)	0 (0.0)	0 (0.0)	0 (0.0)

139 patients were identified who had been treated with an antipsychotic drug either during hospital admission or in the 52 weeks prior to admission. Table indicates characteristics of groups based on different psychiatric diagnosis at time of discharge. FGA = First Generation Antipsychotic; CPZ = Chlorpromazine.

Risperidone was the most commonly prescribed antipsychotic at the time of discharge from the hospital (34% of patients) followed by quetiapine (32% of patients), and olanzapine (9%) (Table 2). The first generation drugs chlorpromazine, haloperidol, loxapine, methotrimeprazine, perphenazine, pimozide, and the second generation drug ziprasidone accounted for only 7 patients, indicating infrequent use of first generation drugs and the low metabolic risk second generation antipsychotic ziprasidone. There were no prescriptions of clozapine prior to hospital admission, and only one for aripiprazole following admission (aripiprazole was approved in Canada in 2009). Drug dosing, based on chlorpromazine equivalent doses, differed significantly between the antipsychotic drugs [F(_5_,_111_) = 8.27, p < 0.0001)]. Amongst the three most commonly prescribed drugs, doses of risperidone were conspicuously lower than for olanzapine or quetiapine.

**Table 2:** Characterization of study population receiving treatment with an antipsychotic medication

	**Risperidone**	**Olanzapine**	**Quetiapine**	**Ziprasidone**	**Aripiprazole**	**FGAs**	**No medication**
Subjects (M/F)	47 (35/12)	13 (6/7)	44 (29/15)	2 (2/0)	1 (0/1)	5 (3/2)	39 (22/17)
Mean Age (SD)	11.54 (3.13)	15.16 (2.12)	13.31 (2.97)	14.63 (0.36)	16.77	15.22 (1.16)	11.88 (3.27)
Mean Dose mg (SD)	1.18 (0.78)	10.77 (4.83)	196.02 (175.58)	30.00 (14.14)	7.50	-	-
CPZ Equiv mg (SD)	58.78 (39.09)	215.38 (96.58)	261.36 (234.10)	50.00 (23.57)	100.00	119.29 (111.33)	-
On-Label Use (% of total on drug)	11 (23.4)	0 (0.0)	0 (0.0)	0 (0.0)	1 (100.0)	0 (0.0)	-
Off-Label Use (% of total on drug)	36 (76.6)	13 (100.0)	44 (100.0)	2 (100.0)	0 (0.0)	5 (100.0)	-
Titrated Switch Pre Hosp (% of switches)	1 (3.1)	2 (6.3)	0 (0.0)	0 (0.0)	0 (0.0)	1 (3.1)	2 (6.3)
Titrated Switch Post Hosp (% of switches)	1 (3.1)	2 (6.3)	2 (6.3)	0 (0.0)	1 (3.1)	0 (0.0)	0 (0.0)
Immediate Switch Pre Hosp (% of switches)	0 (0.0)	0 (0.0)	2 (6.3)	0 (0.0)	0 (0.0)	0 (0.0)	2 (6.3)
Immediate Switch Post Hosp (% of switches)	0 (0.0)	0 (0.0)	0 (0.0)	0 (0.0)	0 (0.0)	0 (0.0)	0 (0.0)
Gap Switch Pre Hosp (% of switches)	0 (0.0)	2 (6.3)	3 (9.4)	0 (0.0)	0 (0.0)	0 (0.0)	3 (9.4)
Gap Switch Post Hosp (% of switches)	0 (0.0)	0 (0.0)	0 (0.0)	0 (0.0)	0 (0.0)	0 (0.0)	0 (0.0)
Overlapped Switch Pre Hosp (% of switches)	3 (9.4)	1 (3.1)	4 (12.5)	0 (0.0)	0 (0.0)	0 (0.0)	2 (6.3)
Overlapped Switch Post Hosp (% of switches)	0 (0.0)	0 (0.0)	1 (3.1)	0 (0.0)	0 (0.0)	0 (0.0)	0 (0.0)

139 patients were identified who had been treated with an antipsychotic drug either during hospital admission or in the 52 weeks prior to admission. Table indicates characteristics of groups based on different antipsychotic drug treatments at time of discharge. FGA = First Generation Antipsychotic; CPZ = Chlorpromazine.

Changes to antipsychotic medication were relatively common in the year before hospital admission, and this frequency increased considerably following admission (Table 3). Of the 139 patients who received antipsychotic prescriptions in the year prior to admission, 25 patients switched between antipsychotic medications at some point during the year prior to their hospital admission, 1 of these patients switched antipsychotic medications twice, and 6 different patients switched antipsychotics following hospital admission for a total of 31 patients switching antipsychotics 32 times. Also, 99 unique patients started an antipsychotic prescription a total of 144 times, and 63 patients terminated an antipsychotic prescription a total of 90 times in the 12 months prior to hospital admission. Following admission, a total of 15 patients started antipsychotics 23 times, and 11 patients stopped antipsychotics 14 times. Many of the switches recorded (40.6%, 13 of 32) were in patients changing from risperidone to quetiapine. Switches from risperidone to olanzapine, quetiapine to risperidone, and quetiapine to olanzapine each occurred in 4 (12.5%) patients. Of the other switches that were performed, haloperidol, aripiprazole, and chlorpromazine were involved in only two switches, and loxapine was involved in a single switch.

**Table 3:** Numbers of patient-weeks as well as the number of starts, stops, and switches were compared

	**Number of weeks**	**Number of switches**	**Switches per year**	**Number of starts**	**Starts per year**	**Number of stops**	**Stops per year**
Before Admission	4321	26	0.313	144	1.733	90	1.083
After Admission	247.7	6	1.260	23	4.828	14	2.939

Starts were defined as any new antipsychotic prescription following a period of at least 3 weeks with no antipsychotic prescriptions; similarly stops were defined as the discontinuation of antipsychotic prescription for at least three weeks. Switches were defined as a new antipsychotic medication started within three weeks (before or after) the termination or beginning of titrating down to termination of another antipsychotic prescription. Frequencies were compared using a Chi-squared test, and all changes were significant.

**Figure 1:**
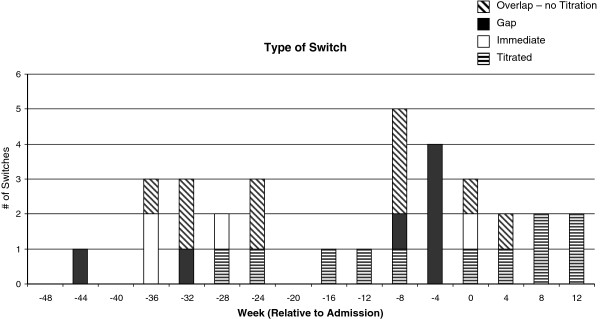
**Distribution and type of switch between antipsychotics made in 52-weeks prior to hospital admission.** Titrated switches were defined as any switch involving either a gradual reduction in dose of the old antipsychotic or a gradual increase in the new antipsychotic within 3 weeks of starting the second drug. Immediate switches were made with no overlap between the two drugs, and no titration. Gap switches involved the termination of one medication with a period of less than three weeks where there were no records of antipsychotic prescriptions prior to starting a different antipsychotic. Overlapped switches involved the two drugs being prescribed concurrently for a period of less than three week with no reduction of dose in the initial antipsychotic, nor a gradual increase in dose in the second drug.

The average maintenance doses were also analyzed for the drugs given before and after all switches. Equivalent dosing of risperidone, olanzapine and quetiapine did not differ significantly before or after the switch with the exception of a single patient who was receiving a maintenance dose of 1000 mg quetiapine and switched to 10 mg of olanzapine (Table 4). All doses before and after switches were also converted to chlorpromazine equivalent doses, as previously [31,32]. Chlorpromazine equivalent doses did not change significantly following the switch. Many of the switches (11 of 32) were clearly titrated, however switches with no sign of titration or a small gap (less than 3 weeks) were also evident (4 of 32 and 7 of 32 respectively), and tended to occur with a higher frequency closer to admission (Figure 1). There were also a notable proportion of switches where the two drugs were prescribed concurrently for a short period with no tapering of either dose (10 of 32 switches).

**Table 4:** Average, minimum, and maximum maintenance doses for antipsychotics

**Prior to switch**				**Following switch**			
**Drug**	**Average**	**Minimum**	**Maximum**	**Drug**	**Average**	**Minimum**	**Maximum**
Risperidone (n = 19)	1.24	0.25	2.5	Risperidone (n = 5)	1.2	0.5	4.0
Olanzapine (n = 3)	10	5	15	Olanzapine (n = 9)	9.17	5	15
Quetiapine (n = 8)	168.75	25	1000	Quetiapine (n = 13)	95.8	37.5	150

Both prior to switching, and following a switch between antipsychotic medications. The doses (mg) are largely similar before and after a switch of antipsychotic medication.

## Discussion

In the present study, we characterized a number of different variables related to antipsychotic drug switching in youth, including frequency of switches, antipsychotic drugs involved and method of switching prior and during admission to a tertiary care psychiatric hospital. The population was heterogeneous in terms of diagnoses, with 63% of all patients having a diagnosis of an anxiety disorder, autism or ADHD at the time of discharge. Dosing in patients treated with risperidone at time of discharge was significantly lower than in patients treated with the alternate second generation antipsychotic drugs olanzapine or quetiapine. This likely reflects the more selective use of risperidone for disruptive behavior and/or anxiety than the other antipsychotics which are preferred for treating the symptoms of psychosis or bipolar disorder.

The frequency of switching in children and adolescents in this cohort was similar to reported frequencies in adults. The rate of 0.31 switches per year of therapy in the community is very close to the rate of 29.5% that was found by Nyhuis et al. [20] in a prospective adult cohort (n = 648), although somewhat lower than the rate of 42% reported by Covell et al. [24] in a chart review of 400 adults with schizophrenia. Whether this difference with the latter study may be due to the age of the current population or use of antipsychotics for disorders primarily other than psychosis remains unknown. Following admission to the B.C. Children’s Hospital, modifications to antipsychotic therapy were made more frequently. This is perhaps not unexpected, as any patient experiencing an exacerbation of their symptoms may tend to be admitted and switch antipsychotics due to a lack of efficacy. However, it is unclear whether the increased switch frequency following admission may also result from differences in prescribing practices between hospital and community physicians, including general practitioners [6].

It is notable that a large proportion of the switches (13, 40.6%) were made from risperidone to quetiapine. A limitation of the current study is that we were not able to ascertain the physician’s reasons for switching antipsychotic drugs. Thus, we do not know whether these switches were related to a lack of efficacy or an intolerance to drug side-effects. For example, risperidone has been associated with equal if not greater efficacy in controlling psychosis in youth [33]. While metabolic side-effects are generally similar between the two drugs [34], risperidone has been associated with higher rates of extrapyramidal symptoms, akathisia and elevated prolactin levels than quetiapine [35,36]. A large proportion of switches from risperidone to quetiapine would therefore be consistent with a lack of drug tolerance in patients, but prospective studies are needed to confirm such hypotheses. The similar levels of dosing before and after antipsychotic switches suggest that the new drug started following a switch did not tend to be effective at lower doses than the drug that was being discontinued. Conversely, the lack of a significant increase in dosing following the switch suggests a level of consistency in dose in terms of chlorpromazine equivalent doses on the part of physicians.

When analyzing the patterns of switching, it was clear that no one particular type of drug switch strategy was predominant. Titrated switches accounted for approximately one third of all switches, while “abrupt” switches accounted for another third, and the final third was evident as concurrent administration of full doses of two drugs for a period followed by abrupt termination of the initial drug. We found no evidence for other types of switches, including the “plateau cross-taper switch” [29,30] (i.e. gradual start of new antipsychotic, followed by treatment with full dose of both drugs, followed by tapering of the original antipsychotic). However, given the modest number of total switches out of a total of 4818 weeks of antipsychotic drug prescription, it is likely that additional strategies for switching antipsychotic drugs would have been evident with a larger sample size. It has been suggested that specific switching strategies may be best suited to certain antipsychotic drug combinations. For example, Buckley and Correll suggested that the plateau cross-titration switch may be preferable for a switch to a drug with a long half-life, such as aripiprazole [29]. We did not find strong evidence for specific patterns of drug switches with specific antipsychotic drug combinations, but again this may reflect the need to reproduce these findings in a considerably larger dataset. However, we did note a greater proportion of titrated switches following admission to hospital compared to prior to admission. This may reflect the high level of expertise and knowledge about antipsychotic drug use and drug titration at the tertiary care facility compared to community settings.

By examining the data on switching longitudinally in the year prior to admission to the hospital, we were able to determine that the frequency of all switches–particularly those without any kind of titration–was clearly higher approaching hospital admission. This finding is open to interpretation. It seems most likely that this reflects a worsening of symptoms which remain refractory to drug treatment, ultimately leading to hospitalization, with switches occurring as an attempt to improve clinical efficacy. While inopportune antipsychotic drug switching can cause adverse effects [25], it would be unlikely for these effects to result in hospitalization.

## Conclusions

In summary, to our knowledge the present findings describe for the first time the frequencies of different patterns of antipsychotic drug switching techniques used in youth with a heterogeneous diagnostic background. Three distinct patterns of switches occurred with similar frequency. Less common switching patterns may also be present in the larger population, as well as switches associated with specific antipsychotic drug combinations. However, observation of these may require the use of larger sample sizes from major administrative databases. Nevertheless, the present study has indicated the feasibility of studying such switches retrospectively using non-chart based procedures, which may be useful for understanding how switching occurs at a larger scale. Effective antipsychotic drug switching remains an important concern, and suboptimal switching may contribute to both patient discomfort and lack of drug adherence. A better understanding of how most antipsychotic drugs switches happen may be one of the first steps to addressing this amenable issue.

## Competing interests

Dr Procyshyn is a paid consultant and is on the speaker’s bureau for AstraZeneca, Bristol-Myers Squibb, Janssen, Otsuka, Pfizer, and Sunovion. Dr Barr has received grants from Bristol-Myers Squibb and advisory fees from Roche. Dr Elbe, Mr Linton and Mr Lee declare no relevant competing interests.

## Authors’ contributions

DL and LHNL acquired and transcribed the data, and developed and analyzed the database. DE and AMB analyzed and interpreted the data. RMP, DL and AMB wrote the manuscript. All authors approved the final version of the manuscript.

## Pre-publication history

The pre-publication history for this paper can be accessed here:

http://www.biomedcentral.com/1471-244X/13/248/prepub
